# Pharmacokinetics and pharmacodynamics of intravenous and inhaled fluticasone furoate in healthy Caucasian and East Asian subjects

**DOI:** 10.1111/bcp.12263

**Published:** 2014-04-22

**Authors:** Ann Allen, Joanne Bal, Anne Cheesbrough, Melanie Hamilton, Rodger Kempsford

**Affiliations:** 1 GlaxoSmithKline R&DStevenage, Hertfordshire, SG1 2NY, UK; 2 GlaxoSmithKline R&DStockley Park, Middlesex, UB11 1BT, UK; 3 GlaxoSmithKline R&DWare, Hertfordshire, SG12 0DP, UK

**Keywords:** Caucasian, East Asian, fluticasone furoate, healthy subjects, pharmacodynamics, pharmacokinetics

## Abstract

**AIM:**

The aim of the study was to evaluate the pharmacokinetics (PK) of inhaled and intravenous (i.v.) fluticasone furoate (FF) in healthy Caucasian, Chinese, Japanese and Korean subjects.

**METHOD:**

This was an open label, randomized, two way crossover study in healthy Caucasian, Chinese, Japanese and Korean subjects (*n* = 20 per group). Inhaled FF (200 μg for 7 days, then 800 μg for 7 days from a dry powder inhaler [DPI]) was administered in one treatment period and i.v.FF (250 μg infusion) in the other. FF PK and serum cortisol (inhaled 200 μg only) were compared between the ethnic groups using analysis of variance. P450 CYP3A4 activity and safety were also assessed.

**RESULTS:**

Ethnic differences in i.v. FF PK were accounted for by body weight differences. CYP3A4 activity was similar across the groups. Higher FF systemic exposure was seen following inhaled dosing in Chinese, Japanese and Korean subjects compared with Caucasian subjects. Absolute bioavailability was greater (36%–55%) in all East Asian groups than for Caucasian subjects following inhaled FF 800 μg. Deconvolution analysis suggested inhaled FF resided in the lung of East Asian subjects longer than for Caucasians (time for 90% to be absorbed [*t*90]: 29.1–30.8 h *vs.* 21.4 h). *In vitro* simulation method predicted comparable delivered lung dose across ethnic groups. Serum cortisol weighted mean was similar between Caucasians and Chinese or Koreans, while in Japanese was on average 22% lower than in Caucasians. All FF treatments were safe and well tolerated.

**CONCLUSION:**

Modestly higher (<50%) FF systemic exposure seen in East Asian subjects following inhaled dosing was not associated with a clinically significant effect on serum cortisol, suggesting that a clinical dose adjustment in East Asian subjects is not required.

**WHAT IS ALREADY KNOWN ABOUT THIS SUBJECT**

- Fluticasone furoate (FF) is in development in combination with vilanterol for the treatment of asthma and chronic obstructive pulmonary disease (COPD).
- Inhaled FF exhibits linear, time-independent pharmacokinetics (PK) with rate-limited absorption from the lung and is predominantly metabolized by CYP3A4.
- The majority of early PK data had been generated in predominantly Caucasian subjects.

**WHAT THIS STUDY ADDS**

- The impact of East Asian ethnicity on the PK of FF has been characterized.
- Higher (<50%) FF systemic exposure in East Asian subjects following inhaled dosing was not associated with a clinically significant effect on serum cortisol, suggesting that clinical dose adjustment in East Asian subjects is not required.

## Introduction

Fluticasone furoate (FF), an inhaled corticosteroid (ICS) with potent glucocorticoid activity, is under development in combination with vilanterol (VI), a potent, inhaled, long acting β_2_-adrenoceptor agonist, for use as a once daily inhaled treatment for asthma and chronic obstructive pulmonary disease (COPD). FF monotherapy is also being developed as inhaled treatment for asthma. The pharmacokinetic (PK), pharmacodynamic (PD) and safety profiles of FF/VI when delivered simultaneously from the dry powder inhaler (DPI) have been described in healthy subjects as well as in patients with asthma and COPD [1,2]. FF/VI has shown favourable safety and tolerability profiles in these subjects [1,2] with little evidence of effects of clinical concern that have previously been reported for ICSs (decreased serum cortisol) [3,4] or long-acting β_2_-adrenoceptor agonists (hypokalaemia, hyperglycaemia and tachycardia) [5,6]. In addition, once daily administration of FF/VI was effective at improving lung function in patients with COPD [7,8] or asthma [9–11] at doses of FF/VI 100/25 μg and 200/25 μg.

Cross-study evaluation of early PK data suggested that FF systemic exposure might be higher in Japanese subjects than in Caucasians (data on file: GlaxoSmithKline R&D, Stevenage, SG1 2NY, UK, [ann.allen@gsk.com]). FF is metabolized primarily by ester hydrolysis via CYP3A4, leading to formation of the 17-carboxylic acid a pharmacologically inactive metabolite [12], The activity of CYP3A4 *in vivo* is essentially similar in subjects of Japanese, Korean, Chinese and Caucasian ancestry [13] and therefore is unlikely to contribute to the apparent differences seen across studies. This study was conducted to evaluate formally this potential difference in FF PK between Japanese and Caucasian subjects. Chinese and Korean subjects were included to determine whether any differences in Japanese subjects, if seen, were applicable to other East Asian ethnic groups. This study evaluated the PK of FF (200 μg and 800 μg repeat dose for 7 days) administered from a two-strip dry powder device and when given as a single intravenous (i.v.) dose (FF 250 μg) in healthy Japanese, Korean and Chinese subjects and Caucasian subjects. Pharmacodynamics (PD) (serum cortisol, pharyngometry), P450 CYP3A4 activity and safety were also assessed.

FF PK were evaluated after repeat administration of FF 200 μg, since this was likely to be the upper clinical dose. In addition, evaluation of the potential for systemic PD effects (serum cortisol) associated with higher FF systemic exposure in East Asian subjects were also conducted at this dose. The relationship between cortisol suppression and FF AUC is well characterized and therefore, the objective of the serum cortisol assessment in this study was to assess potential for effect at the clinical dose rather than at the supra-therapeutic dose. At the 800 μg supra-therapeutic dose cortisol suppression would have been anticipated for all ethnic groups. Based on previous data, it was anticipated that the PK data following FF 200 μg would be censored due to concentrations falling below the lower limit of quantification and possibly lead to inconclusive data for the ethnic group comparisons. Therefore a supra-therapeutic dose of FF 800 μg (single and repeat dose) was also studied to ensure robust PK data were obtained for the ethnic group comparisons. The study included i.v. administration of FF 250 μg to determine total plasma clearance (CL) and steady-state volume of distribution (*V*_ss_) of FF, the absorption time from the lung and absolute bioavailability of inhaled FF. Plasma 4-β-hydroxy-cholesterol and the cortisol : 6-β-hydroxy cortisol ratio were used to assess and compare CYP3A4 activity in these ethnic groups. A further (exploratory) objective to the study was an evaluation of dose emission attributes of FF to gain an understanding of the relationship between dose (in terms of mass) and its particle size distribution and PK parameters.

## Methods

### Study design and subjects

The trial was conducted in compliance with Good Clinical Practice and the Declaration of Helsinki. The investigators obtained institutional review board approvals (Bellberry Limited, 229 Greenhill Road, Dulwich, SA, Australia 5065) for the study protocol (HZA113477; NCT01000597), and the study was conducted at GlaxoSmithKline Medicines Research Unit, Prince of Wales Hospital, Randwick, NSW, Australia. All subjects gave their written informed consent before participating in the trial.

**Figure 1:**
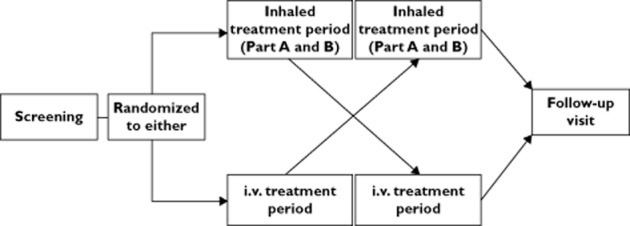
Study design schematic

This was an open label, randomized, single and repeat dose, two way crossover study in healthy male and female non-smoking subjects of Caucasian, Japanese, Korean and Chinese ethnic origin (see Online Supporting Information for the definition used for each ethnic origin). Each subject (*n* = 20 per group) was to participate in two treatment periods (Figure 1). The randomization schedule was generated using validated internal software, using a block size of four and stratifying by ethnic group. The inhaled treatment period was conducted in two parts, part A and part B. In part A the FF 200 μg dose was given as two inhalations of FF 100 μg via the ELLIPTA™^1^ DPI in the morning. There was a minimum 24 h washout period (maximum 7 days) between the 24 h PK sample following the day 7 dose in part A, and the first dose on day 1 in part B. In part B the FF 800 μg dose was given as two inhalations of FF 400 μg via the DPI in the morning. Full details of the study/clinic visits, the timing of assessments, restrictions related to food, drink and exercise intake, and assessment of treatment compliance are provided in the Online Supporting Information.

For the FF single dose i.v. treatment period (1 ml of 250 μg ml^−1^ FF in 100% propylene glycol administered at a constant rate of infusion over 20 min using a syringe and pump), each subject attended the unit on the morning of day −1. They then remained in the unit until completion of all assessments at 24 h post-dose on day 2. Subjects returned to the clinical unit for their 32 and 48 h post-dose PK sampling. The inhaled and i.v. treatment periods were separated by a washout period of at least 7 days and no more than 14 days.

Subjects attended a post-study follow-up visit within 7–14 days of their last dose. The duration of the study was approximately 10 weeks for each subject.

### Pharmacokinetic endpoints

PK endpoints included plasma concentrations and derived FF PK parameters on day 7 for FF 200 μg, day 1 and day 8 for FF 800 μg, and day 1 for FF 250 μg i.v.. Venous blood samples (approximately 4 ml) for analysis of plasma drug concentrations were collected in KEDTA tubes pre-dose and at scheduled times that varied depending on the treatment (see Online Supporting Information for full details). The blood samples were put on ice until centrifugation at 2000 *g* for approximately 10 min at 4°C. Following centrifugation, supernatant plasma was transferred into 1.8 ml matrix screw capped polypropylene tubes and frozen. All samples were stored at −20°C or colder until shipment for analysis at Worldwide Bioanalysis Group, Ware, UK.

Plasma samples were analysed for FF (150 μl of plasma) by solid phase extraction using a validated analytical method based on solid phase extraction (with [^13^C ^2^H_3_]-GW685698 as the internal standard) followed by high performance liquid chromatography with tandem mass spectrometry using an Applied Biosystems API-5000 (Applied Biosystems/MDS Sciex,Foster City, USA). The validation range of the assay was 10–1000 pg ml^−1^ for FF and the lower limit of quantification for FF was 10 pg ml^−1^. Within-run precision, between-run precision and bias were all ≤14.3%. Further details of the plasma sample analyses are included in the Online Supporting Information.

### Safety endpoints

Safety endpoints included adverse events (AEs) collected over the duration of the study period, laboratory safety tests (haematology, clinical chemistry and urinalysis) conducted at screening, prior to each treatment period and at follow-up, vital signs, 12-lead electrocardiography (ECG) and peak expiratory flow rate (PEFR) conducted at screening, at intervals during each treatment period and at follow-up and Holter monitoring conducted at screening only. Clinical laboratory tests were analysed locally by the site for each study.

Venous blood samples (approximately 2 ml) for analysis of serum cortisol concentrations were collected pre-dose and at 1, 2, 3, 4, 6, 8, 10, 12, 16 and 24 h after the start of inhaled FF 200 μg on day 7. Further details of the serum cortisol analyses are summarized in the Online Supporting Information.

Single blood samples for plasma 4-β-hydroxy-cholesterol and 24 h urine collections for cortisol : 6-β-hydroxy cortisol ratio were collected to assess CYP3A4 activity.

### Inhalation and pharyngometry profiling

These were exploratory assessments conducted at the time of the first and last dose of the repeat inhalation treatment periods for FF 200 μg and FF 800 μg to develop an *in vitro* simulation method. Pharyngometry profiles were conducted using acoustic reflectance (Pharyngometer, Eccovision, Sleep Group Solutions, Miami Beach, CA, USA) to estimate mouth and throat size and inhalation profiles were recorded to an Inhalation Profile Recorder (GlaxoSmithKline, Ware, UK) using two methods: method one recorded pressure drop *vs.* time profiles and method two recorded flow rate *vs.* time and breath hold profiles using respiratory inductive plethysmography belts. Inhalation and breath hold profiles were recorded during the inhaled treatment period only on days −1, 1, 7 and 8 (part A) and days −1, 1, 8 and 9 (part B). Profiles recorded using only the respiratory inductive plethysmography belts coincided with when the subject inhaled through the inhaler appropriate to the treatment period.

Replication of selected pressure drop *vs.* time inhalation profiles using an Electronic Lung (*in vitro* Breathing Simulator, GlaxoSmithKline, Ware, UK), and coated anatomical throat cast, provided modelling and prediction of total emitted dose, ex-throat dose (ETD) and mass <2 μm of FF when delivered from the DPI [14].

### Pharmacokinetic analysis

PK analyses of plasma FF concentration–time data following inhaled and i.v. administration were conducted using non-compartmental Model 200 (for extravascular administration) and Model 202 (for constant infusion), respectively, of WinNonlin Professional Edition Version 5.2 (Pharsight Corporation, Mountain View, CA, USA). PK variables for all treatments were calculated as follows: maximum concentration (*C*_max_) and time to maximum concentration (*t*_max_) were derived directly from plasma concentration–time profiles, the slowest disposition rate constant (λ_z_) was calculated by log-linear regression of the terminal portion of the concentration–time profiles where there were sufficient data, terminal elimination half life (*t*_1/2_) was calculated as 0.693/λ_z_, area under the concentration–time curve from 0–24 h (AUC(0,24 h)) and area under the concentration–time curve from time zero to time of last quantifiable concentration (AUC(0,*t*_last_)) were calculated using the linear trapezoidal rule for intervals where the concentration data were increasing, and the logarithmic trapezoidal rule for intervals where the concentration data was decreasing, and then extrapolated to infinity using λ_z_ to obtain the area under the concentration–time curve from time zero extrapolated to infinity (AUC(0,∞)). Mean absorption time (MAT) was estimated as the difference between mean residence time following inhaled dosing and mean residence time following i.v. dosing. For i.v. treatment only, CL and *V*_ss_ were also determined.

FF concentration–time data (800 μg inhaled) were subjected to deconvolution analysis using WinNonlin Pro (Version 5.2). The micro-constants describing distribution and elimination were obtained by fitting an appropriate infusion model to the time–concentration data following i.v. dosing of FF using WinNonlin Pro [Version 5.2]. The absorption rate constant (*k*_a_) was obtained by fitting a mono-exponential function to the percent remaining to be absorbed *vs.* time data visually assessed to lie on the linear portion of the semi-logarithmic plots. The absorption half-life (*t*_1/2_,_abs_) was calculated as the ratio of ln2/*k*_a_ and this was used to calculate the time for 90% to be absorbed (*t*90).

### Statistical analysis

AUC(0,*t*_last_), AUC(0,24 h), AUC(0,∞) (day 1 only), *C*_max_, *t*_1/2_, MAT (inhaled dosing only), CL (i.v. only) and *V*_ss_ (i.v. only) were compared between ethnic groups (each East Asian group compared with Caucasian) for each treatment group. With the exception of AUC(0,∞) and MAT, comparisons for inhaled treatments were performed using a mixed model analysis of variance with period, treatment, ethnic group and ethnic group by treatment interaction fitted as fixed effects and subject as a random effect. Comparisons for the i.v. treatment and for AUC(0,∞) and MAT from the inhaled treatments were performed using analysis of variance model with period and ethnic group as fixed effects.

Absolute bioavailability was compared between ethnic groups using a mixed model analysis of variance with period, treatment, ethnic group and ethnic group by treatment interaction fitted as fixed effects and subject as a random effect.

Accumulation ratios for FF 800 μg were determined from AUC and *C*_max_ on days 1 and 8. Mixed effects models were fitted with fixed effect terms for day, ethnic group and day by ethnic group interaction. Subject was treated as a random effect in the model.

Weighted mean 24 h serum cortisol for FF 200 μg was compared between ethnic groups (each East Asian group compared with Caucasian) using analysis of variance with day −1 value (baseline) period and ethnic group fitted as fixed effects.

## Results

### Study population

Eighty subjects (20 per group) were enrolled, and 18 Caucasian, 20 Chinese, 19 Japanese and 20 Korean subjects completed the study as planned. Two Caucasian subjects were withdrawn, one due to an AE and one at the discretion of the Investigator. The onset of the AE occurred pre-treatment and was considered mild and unrelated to treatment. This subject received a single i.v. dose of FF 250 μg prior to withdrawal, and did not take part in the inhaled FF dosing sessions. One Japanese subject was withdrawn due to a protocol deviation. All withdrawals were considered unrelated to study treatment. Subject demographics are shown in Table 1. As would be expected, the Chinese, Japanese and Korean subjects had lower body weight and height compared with Caucasian subjects, although there was some overlap in the ranges. Total lung capacity was also lower for Chinese, Japanese and Korean subjects than for Caucasian subjects. There was no evidence for a difference in CYP3A4 activity between the ethnic groups as measured by both urine cortisol : 6-β-hydroxy cortisol ratio and plasma 4-β-hydroxy-cholesterol (Table 1).

**Table 1:** Summary of subject disposition and demographic characteristics

Number of subjects	Ethnic group			
	Caucasian	Chinese	Japanese	Korean
** Subjects randomized, *n***	20	20	20	20
** Subjects completed, *n***	18 (90)	20 (100)	19 (95)	20 (100)
** Mean age years (range)**	27.1 (21–40)	27.7 (20–49)	30.3 (21–59)	25.9 (22–30)
** Male, *n* (%)**	17 (85)	10 (50)	15 (75)	19 (95)
** Mean BMI, kg m^−2^ (range)**	24.9 (20.2–29.4)	21.7 (18.6–24.8)	21.4 (18.8–24.9)	22.4 (18.7–24.9)
** Mean height, cm (range)**	179 (161–193)	171 (156–184)	170 (155–182)	174 (157–183)
** Mean weight, kg (range)**	79.7 (59.4–96.0)	63.5 (48.8–81.4)	61.6 (49.0–74.5)	67.9 (56.0–80.2)
**CYP3A4 activity**				
** Ratio urine cortisol : 6-β-hydroxy cortisol Geometric mean (CVb%)**	0.137 (33.8)	0.185 (45.9)	0.140 (41.5)	0.192 (40.4)
** Plasma 4-β-hydroxy-cholesterol (μg l^−1^) Geometric mean (CVb%)**	54.4 (28.8)	57.6 (23.6)	61.5 (28.7)	50.7 (23.2)
**Pulmonary function test (screening)**				
** FEV_1_ (l) mean (95% CI)**	4.56 (4.19, 4.92)	3.79 (3.43, 4.15)	4.06 (3.79, 4.33)	4.44 (4.18, 4.70)
** PEFR (l min^−1^) mean (95% CI)**	601.7 (554.5, 649.0)	515.3 (470.3, 560.2)	597.6 (546.8, 648.4)	660.7 (616.9, 704.5)
** Total lung capacity (l)**	7.29 (6.71, 7.87)	5.96 (5.31, 6.60)	6.41 (5.99, 6.83)	6.54 (6.08, 7.01)
** Mean (95% CI)**				

BMI, body mass index; CI, confidence interval; CYP, cytochrome P450; CVb%, between-subject coefficient of variation; FEV_1_, forced expiratory volume in 1 s; PEFR, peak expiratory flow rate.

### Pharmacokinetic endpoints

Eighteen Caucasian, 20 Chinese, 19 Japanese and 20 Korean subjects provided complete datasets for PK evaluation. One Caucasian subject had unusually low FF plasma concentrations following i.v. FF 250 μg compared with all other subjects who received i.v. FF 250 μg in this study. This resulted in an estimate of AUC(0,∞) that was 78% lower than the geometric mean and 76% lower than the lower limit of the 95% confidence interval (CI) for Caucasian subjects (following exclusion). The results presented exclude data for this subject.

#### Intravenous fluticasone furoate 250 μg

**Figure 2:**
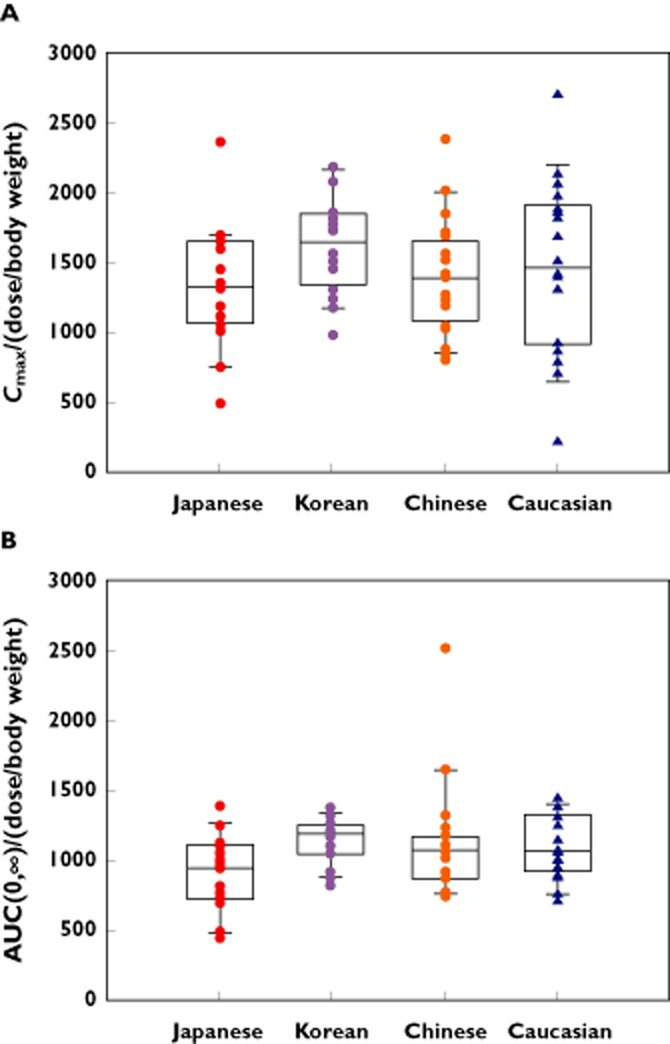
Box and whisker plots for body weight normalized (μg kg^−1^) *C*_max_; (A) and AUC(0,∞) following after administration of i.v. FF 250 μg; (B). AUC(0,∞), area under the concentration–time curve from time zero extrapolated to infinity; *C*_max_, maximum concentration; FF, fluticasone furoate

The mean plasma concentration–time profiles following i.v. FF 250 μg were essentially superimposable for all ethnic groups over the entire 48 h post-dose period (Figure S1). Maximum plasma concentrations were generally observed at the end of the 20 min infusion followed by a poly-exponential decline with an average terminal phase elimination half-life of 14–18 h (Table 2). There was some evidence of greater systemic exposure to FF, based on *C*_max_ and AUC(0,∞) for Chinese, Japanese and Korean subjects, when compared with Caucasian subjects (Table 2). However, where differences in *C*_max_ and AUC(0,∞) were observed these were less than 44% and 25%, respectively. These differences were consistent with the lower body weights in the Chinese, Japanese and Korean populations compared with Caucasians and are supported by lack of difference in weight-normalized *C*_max_ and AUC values (Figure 2) and statistical analysis showed there was no evidence for a difference in body weight normalized *C*_max_ and AUC(0,∞) between any Asian ethnic group and Caucasians, apart from *C*_max_ for Korean subjects. The PK parameters describing the inherent PK characteristics of FF (CL, *V*_ss_, *t*_1/2_) following i.v. administration indicated that there were no differences in *V*_ss_ between Caucasian, Chinese, Japanese and Korean subjects. Only the Chinese and Korean subjects showed lower CL (on average <20%) than Caucasian subjects, and the Chinese subjects had a longer *t*_1/2_ (on average 34% greater) than Caucasians (Table 2).

**Table 2:** Summary of fluticasone furoate pharmacokinetic parameters following FF 250 μg single dose i.v. administration and statistical analysis results

Parameter (units)	Caucasian subjects *n* = 19		Chinese subjects *n* = 20		Japanese subjects *n* = 20		Korean subjects *n* = 20		Statistical comparison		
	*n*	Geometric mean (95% CI)	*n*	Geometric mean (95% CI)	*n*	Geometric mean (95% CI)	*n*	Geometric mean (95% CI)	Ethnic group comparison	Ratio of adjusted geometric mean	90% CI of ratio
***C*_max_ (pg ml^−1^)**	18	4224(3278, 5442)	20	5431(4878, 6046)	19	5231(4498, 6083)	20	5972(5400, 6604)	Chinese : Caucasian	1.296	(1.085, 1.548)
									Japanese : Caucasian	1.270	(1.059, 1.522)
									Korean : Caucasian	1.435	(1.201, 1.714)
*t*_max_ (h)*	18	0.33(0.17–0.75)	20	0.33(0.17–0.75)	19	0.33(0.17–0.42)	20	0.33(0.17–0.35)	Chinese : Caucasian	NC	NC
									Japanese : Caucasian		
									Korean : Caucasian		
**AUC(0,∞) (pg ml^−1^ h)**	17	3505(3206, 3832)	19	4347(3855, 4901)	18	3537(3067, 4079)	19	4194(3915, 4493)	Chinese : Caucasian	1.243	(1.099, 1.407)
									Japanese : Caucasian	1.019	(0.897, 1.157)
									Korean : Caucasian	1.204	(1.063, 1.364)
***t*_1/2_ (h)**	17	13.7(10.7, 17.6)	19	18.2(16.1, 20.6)	18	14.7(11.9, 18.2)	19	16.2(15.4, 17.2)	Chinese : Caucasian	1.341	(1.106, 1.625)
									Japanese : Caucasian	1.107	(0.908, 1.349)
									Korean : Caucasian	1.209	(0.996, 1.467)
**CL (l h^−1^)**	17	71.3(65.2, 78.0)	19	57.5(51.0, 64.9)	18	70.7(61.3, 81.5)	19	59.6(55.6, 63.9)	Chinese : Caucasian	0.804	(0.711, 0.910)
									Japanese : Caucasian	0.982	(0.865, 1.115)
									Korean : Caucasian	0.830	(0.733, 0.940)
***V*_ss_ (l)**	17	607(473, 780)	19	652(516, 823)	18	681(510, 910)	19	561(508, 620)	Chinese : Caucasian	1.074	(0.830, 1.389)
									Japanese : Caucasian	1.123	(0.861, 1.464)
									Korean : Caucasian	0.925	(0.713, 1.199)

* Median (range). AUC(0,∞), area under the concentration–time curve from time zero extrapolated to infinity; CI, confidence interval; CL, total plasma clearance; *C*_max_, maximum concentration; *n*, number of subjects for whom pharmacokinetic parameter was derived; NC, statistical comparison not conducted; *t*_1/2_, terminal elimination half-life; *t*_max_, time to maximum concentration; *V*_ss_, steady-state volume of distribution.

#### Inhaled fluticasone furoate 800 μg for 7 days

**Figure 3:**
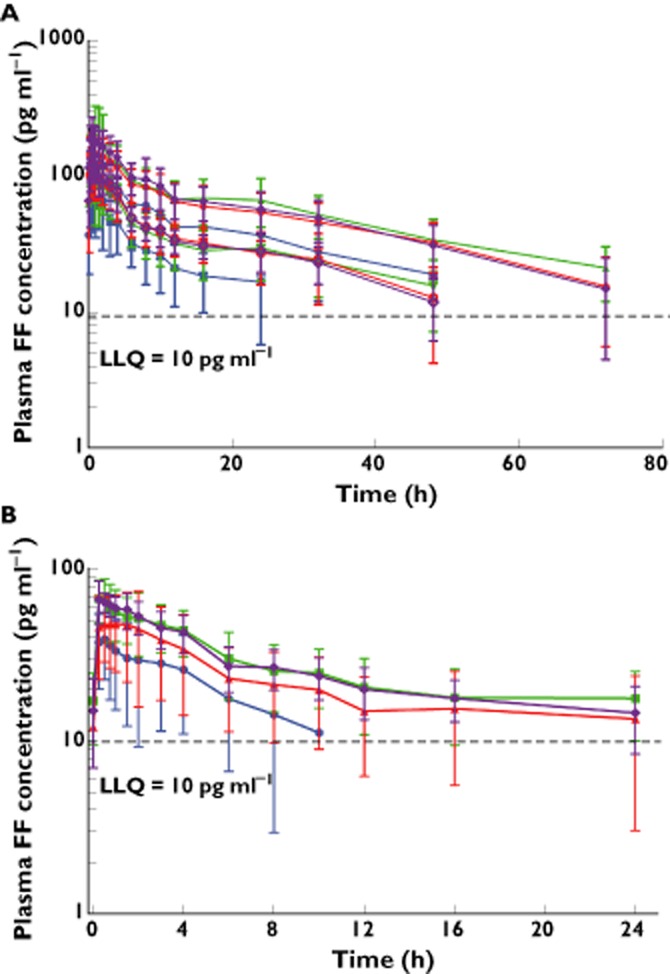
Mean\ ± SD plasma semi-log concentration–time profiles for FF after administration of inhaled FF 800 μg single and repeat dose; (A) and inhaled FF repeat dose 200 μg; (B). FF, fluticasone furoate; LLQ, lower limit of quantification; 3A: (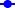) Caucasian (repeat), (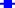) Caucasian (single), (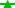) Chinese (repeat), (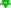) Chinese (single), (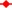) Japanese (repeat), (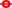) Japanese (single), (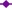) Korean (repeat), and (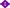) Korean (single). 3B: (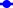) Caucasian, (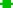) Chinese, (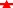) Japanese and (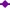) Korean

In all four ethnic groups, maximum plasma concentrations were observed on average at 30–45 min post-dose followed by a poly-exponential decline, and there was evidence of higher FF plasma concentrations in Chinese, Japanese and Korean subjects compared with Caucasian subjects (Figure 3A). Following both single and repeat inhaled administration of FF 800 μg there were consistently higher *C*_max_ and AUC values in all three East Asian groups with geometric mean ratios for *C*_max_ and AUC ranging from 40% to 76% greater and 52% to 75% greater, respectively, compared with the Caucasian group (Table 3). There was also evidence for longer *t*_1/2_s in Chinese and Japanese subjects than Caucasian subjects, although differences were on average <30% (Table 3) and did not result in greater accumulation. Based on the ratio of geometric means for AUC(0,24 h), the observed accumulation was 87%, 110%, 72% and 92% for Caucasian, Chinese, Japanese and Korean subjects, respectively.

**Table 3:** Summary of fluticasone furoate pharmacokinetic parameters following inhaled fluticasone furoate 800 μg repeat dose and statistical analysis results

Parameter (units)	Caucasian subjects *n* = 19		Chinese subjects *n* = 20		Japanese subjects *n* = 20		Korean subjects *n* = 20		Statistical comparison		
	*n*	Geometric mean (95% CI)	*n*	Geometric mean (95% CI)	*n*	Geometric mean (95% CI)	*n*	Geometric mean (95% CI)	Ethnic group comparison	Ratio of adjusted geometric mean	90% CI of ratio
***C*_max_ (pg ml^−1^)**	18	118(99.6, 141)	20	181(146, 225)	20	164(144, 186)	20	206(181, 234)	Chinese : Caucasian	1.542	(1.282, 1.854)
									Japanese : Caucasian	1.404	(1.167, 1.689)
									Korean : Caucasian	1.755	(1.459, 2.111)
*t*_max_ (h)*	18	0.63(0.25–2.00)	20	0.75(0.25–4.00)	20	0.50(0.00–1.00)	20	0.50(0.25–1.50)	Chinese : Caucasian	NC	NC
									Japanese : Caucasian		
									Korean : Caucasian		
**AUC(0,24 h) (pg ml^−1^ h)**	18	1203(947, 1528)	20	1989(1630, 2428)	20	1817(1546, 2136)	20	2093(1841, 2380)	Chinese : Caucasian	1.658	(1.365, 2.013)
									Japanese : Caucasian	1.520	(1.250, 1.848)
									Korean : Caucasian	1.747	(1.438, 2.123)
***t*_1/2_ (h)**	15	25.5(22.6, 28.8)	19	32.5(30.7, 34.5)	19	30.6(27.6, 33.9)	20	27.0(25.2, 28.9)	Chinese : Caucasian	1.283	(1.040, 1.583)
									Japanese : Caucasian	1.255	(1.017, 1.549)
									Korean : Caucasian	1.120	(0.909, 1.379)

* Median (range). AUC(0,∞), area under the concentration-time curve from time zero extrapolated to infinity; CI, confidence interval; CL, total plasma clearance; *C*_max_, maximum concentration; *n*, number of subjects for whom pharmacokinetic parameter was derived; NC, statistical comparison not conducted; *t*_1/2_, terminal elimination half-life; *t*_max_, time to maximum concentration.

#### Inhaled fluticasone furoate 200 μg for 7 days

In all four ethnic groups, maximum plasma concentrations were observed, on average at 30 min post-dose followed by a poly-exponential decline (Figure 3B). Although the data were somewhat censored due to concentrations below the lower limit of quantification (LLQ 10 pg ml^−1^) the FF 200 μg data were consistent with the data at FF 800 μg. Both AUC(0,24 h) and *C*_max_ were consistently higher in the East Asian ethnic groups, by an average of 27%–49% and 37%–78%, respectively, compared with values in the Caucasian group (Table 4).

**Table 4:** Summary of fluticasone furoate pharmacokinetic parameters following inhaled FF 200 μg repeat dose and statistical analysis results

Parameter (units)	Caucasian subjects *n* = 19		Chinese subjects *n* = 20		Japanese subjects *n* = 20		Korean subjects *n* = 20		Statistical comparison		
	*n*	Geometric mean (95% CI)	*n*	Geometric mean (95% CI)	*n*	Geometric mean (95% CI)	*n*	Geometric mean (95% CI)	Ethnic group comparison	Ratio of adjusted geometric mean	90% CI of ratio
***C*_max_ (pg ml^−1^)**	18	41.1(32.8, 51.5)	20	66.9(57.5, 77.9)	20	55.7(46.6, 66.5)	20	72.3(65.9, 79.3)	Chinese : Caucasian	1.637	(1.361, 1.969)
									Japanese : Caucasian	1.373	(1.141, 1.653)
									Korean : Caucasian	1.776	(1.477, 2.137)
*t*_max_(h)*	18	0.50(0.25–3.00)	20	0.50(0.25–4.00)	20	0.50(0.25–4.00)	20	0.50(0.25–2.00)	Chinese : Caucasian	NC	NC
									Japanese : Caucasian		
									Korean : Caucasian		
**AUC(0,*t*_last_) (pg ml^−1^ h)**	18	222(135, 366)	20	588(454, 761)	20	430(317, 584)	20	607(536, 686)	Chinese : Caucasian	NC	NC
									Japanese : Caucasian		
									Korean : Caucasian		
**AUC(0,24 h) (pg ml^−1^ h)**	9	541(440, 666)	18	680(603, 767)	16	561(454, 694)	19	629(566, 698)	Chinese : Caucasian	1.487	(1.190, 1.858)
									Japanese : Caucasian	1.274	(1.015, 1.598)
									Korean : Caucasian	1.439	(1.153, 1.797)

* Median (range). AUC(0,24 h), area under the concentration time curve from time zero to 24 h; AUC(0,*t*_last_), area under the concentration–time curve from time zero to time of last quantifiable concentration; CI, confidence interval; *C*_max_, maximum concentration; *n*, number of subjects for whom pharmacokinetic parameter was derived; NC, statistical comparison not conducted; *t*_max_, time to maximum concentration.

#### Absolute bioavailability of inhaled fluticasone furoate

Absolute bioavailability values for each of the inhaled FF treatments were estimated separately for each ethnic group. Geometric mean values for absolute bioavailability of inhaled FF ranged from 10.4% to 20.0% (Table 5). Data at FF 200 μg were less reliable than at FF 800 μg due to larger numbers of subjects in the Caucasian group not reporting AUC(0,24 h) values due to the lower systemic exposure observed in that group. For the FF 800 μg treatment on both day 1 and day 8 there was evidence of higher bioavailability in the three East Asian groups than in the Caucasian group. The most robust inhaled data were observed following FF 800 μg for 7 days where adjusted geometric mean values for absolute bioavailability ranged from 36% to 55% higher in the East Asian populations, compared with the Caucasian group.

**Table 5:** Summary of absolute bioavailability (%) of fluticasone furoate following inhaled administration and statistical analysis results

Treatment	Ethnic group		Geometric mean (95% CI)	Comparison	Ratio of adjusted geometric mean	90% CI of ratio
**FF 200 μg**	Caucasian	8	19.2 (14.6, 25.3)	Chinese : Caucasian	1.279	(0.953, 1.716)
**Day 7**	Chinese	17	20.0 (16.6, 24.0)	Japanese : Caucasian	1.243	(0.918, 1.685)
	Japanese	14	19.2 (15.4, 24.0)	Korean : Caucasian	1.306	(0.974, 1.753)
	Korean	18	18.8 (16.7, 21.1)			
**FF 800 μg**	Caucasian	11	11.2 (7.70, 16.3)	Chinese : Caucasian	1.356	(1.026, 1.791)
**Day 1**	Chinese	19	14.1 (9.70, 20.4)	Japanese : Caucasian	1.887	(1.417, 2.513)
	Japanese	17	19.7 (15.1, 25.7)	Korean : Caucasian	1.421	(1.074, 1.881)
	Korean	19	15.0 (13.5, 16.7)			
**FF 800 μg**	Caucasian	16	10.4 (8.10, 13.4)	Chinese : Caucasian	1.364	(1.045, 1.779)
**Day 8**	Chinese	19	14.3 (11.3, 18.1)	Japanese : Caucasian	1.551	(1.180, 2.039)
	Japanese	18	16.3 (12.5, 21.3)	Korean : Caucasian	1.469	(1.124, 1.920)
	Korean	19	15.4 (13.5, 17.6)			

CI, confidence interval; FF, fluticasone furoate.

#### Absorption pharmacokinetics of inhaled fluticasone furoate

**Figure 4:**
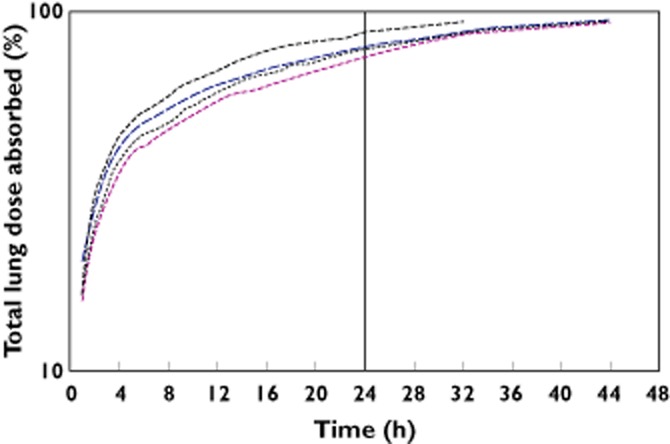
Total FF lung dose absorbed–time profiles after administration of inhaled FF 800 μg single dose estimated from deconvolution analysis. FF, fluticasone furoate. (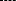) Caucasian, (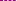) Chinese, (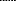) Japanese, and (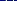) Korean

In all four ethnic groups the *t*_1/2_ following FF 800 μg inhaled administration for 7 days was notably longer than that seen following i.v. dosing (26–33 h *vs.* 14–18 h; Tables 2 and 3). This suggested that the elimination of FF is absorption rate limited following inhaled administration. To investigate this further, the inhaled concentration–time data following FF 800 μg single dose (day 1) were subjected to deconvolution analysis utilizing the Loo-Riegelman model [15], and time-percent remaining to be absorbed was generated. There was evidence of longer MAT in all East Asian ethnic groups, compared with the Caucasian group (9.67–10.6 h *vs.* 5.15 h) and consistent with this, both *t*_1/2,abs_ from the lung and *t*90 for FF were longer in East Asian subjects than in Caucasians (Table 6). Although there were small differences in the rate of absorption of FF between East Asian subjects and Caucasian subjects, FF absorption was essentially complete for all populations by 24 h post-dose (Figure 4).

**Table 6:** Summary of fluticasone furoate absorption pharmacokinetic parameters following inhaled administration of 800 μg single dose

Parameter (units)	Caucasian subjects *n* = 19		Chinese subjects *n* = 20		Japanese subjects *n* = 20		Korean subjects *n* = 20	
	*n*	Geometric mean (95% CI)	*n*	Geometric mean (95% CI)	*n*	Geometric mean (95% CI)	*n*	Geometric mean (95% CI)
**MAT (h)**	18	5.15(2.84, 9.32)	20	10.6(8.72, 12.8)	19	9.67(7.78, 12.0)	20	10.6(9.41, 11.9)
***t*90 (h)**	18	21.4(16.3, 28.1)	20	29.9(23.7, 37.8)	18	29.1(25.2, 33.6)	20	30.8(28.8, 32.9)
***t*_1/2_,_abs_ (h)**	18	8.05(6.02, 10.8)	20	9.21(7.21, 11.7)	18	9.91(8.29, 11.8)	20	10.2(9.44, 10.9)

CI, confidence interval; MAT, mean absorption time; *t*_1/2, abs_, absorption half-life; *t*90, time for 90% to be absorbed.

### Pharmacodynamic endpoints

#### Serum cortisol

**Figure 5:**
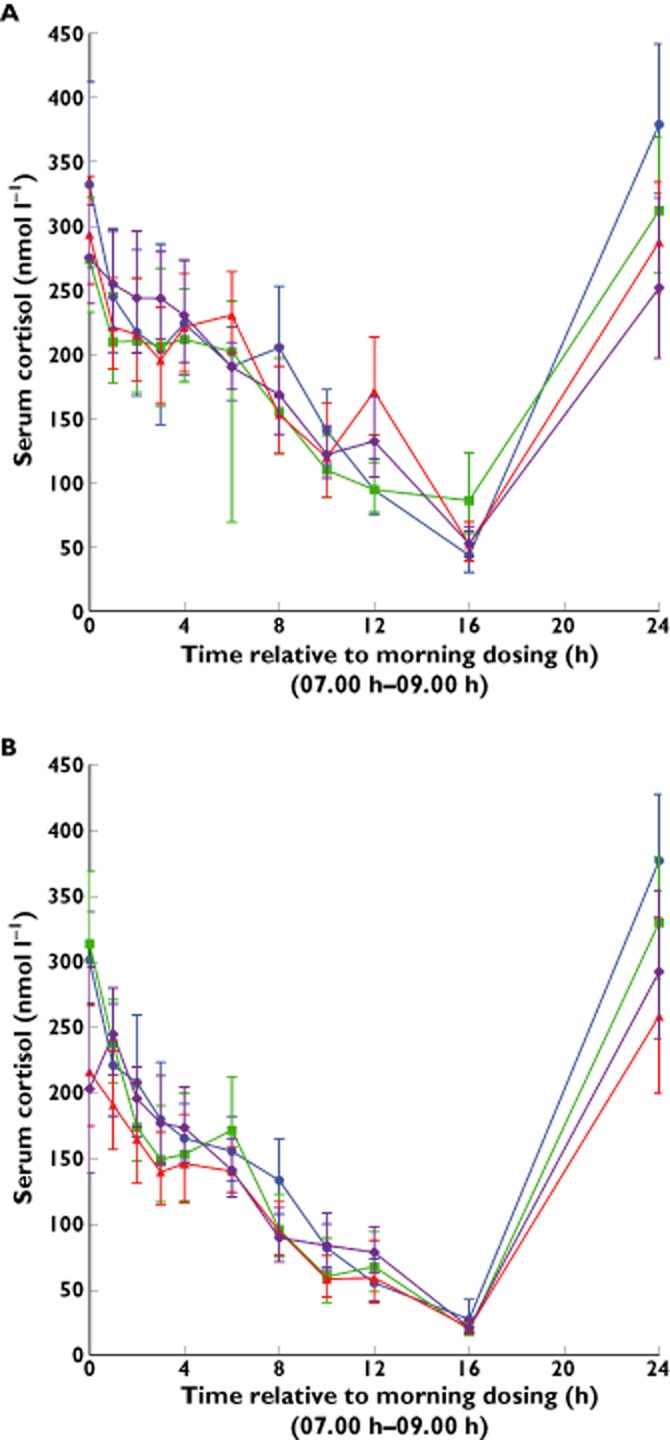
Geometric mean and 95% CI serum cortisol concentration–time profile (0–24 h) on day −1 (pre-FF); (A) and following 7 days repeat inhaled administration of FF 200 μg; (B). CI, confidence interval; FF, fluticasone furoate. (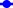) Caucasian, (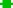) Chinese, (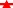) Japanese and (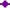) Korean

The diurnal 24 h serum cortisol profile was similar for all ethnic groups both prior to treatment (day −1) and after 7 days FF 200 μg once daily (day 7) (Figure 5). There was no evidence for a difference in 24 h weighted mean serum cortisol between the Chinese or Korean ethnic groups compared with Caucasian subjects. There was on average a 22% lower 24 h serum cortisol weighted mean for Japanese subjects compared with Caucasians which suggested that the extent of cortisol suppression among Japanese subjects was greater than among Caucasian subjects (Figure 5). However, there was still considerable overlap in individual values and 17 of the 20 Japanese subjects had values within the range of values in Caucasian subjects (Figure S2).

### Safety

All FF treatments were safe and well tolerated with no marked quantitative or qualitative differences between the ethnic groups. Few AEs were reported by more than one subject following any treatment within each ethnic group. Fifty-three subjects (66%) reported at least one AE in the study, irrespective of causality. The most frequently reported AE was headache, which occurred in 20 subjects (25%) across treatment regimens and ethnic groups. The rate of AEs and the most frequently reported AEs by treatment regimen and by ethnic group are summarized in Table S1.

One Caucasian subject withdrew due to an AE (anogenital warts). The onset of the AE occurred pre-treatment and was considered mild and unrelated to treatment. All AEs in the study were considered by the Investigator to be of mild or moderate intensity. There were no AEs of severe intensity. There were no ECG, vital sign or clinical laboratory abnormalities of clinical significance in any subject for any treatment.

### Pharyngometry and inhalation profiles

Pharyngometry data provided information on mouth and throat geometry, from lips to larynx. The mean average cross-sectional area was 2.71 cm^2^, 2.89 cm^2^, 2.61 cm^2^ and 2.89 cm^2^ for Caucasian, Chinese, Japanese and Korean subjects, respectively.

The mean peak inspiratory flow rate recorded from pressure drop *vs.* time profiles across time points within the study (post-screening) was 90.1 l min^−1^, 75.5 l min^−1^, 74.5 l min^−1^ and 93.8 l min^−1^ for Caucasian, Chinese, Japanese and Korean subjects, respectively. Height and body mass index differences across the ethnic groups were also thought to influence the inhalation parameters achieved although there was a lot of overlap in both parameters between the groups.

#### Total emitted dose, ex-throat dose and mass <2 μm

Through the use of the *in vitro* model, mean total emitted dose, calculated as the sum of all stages post-inhaler was predicted to be approximately 82% of the nominal dose (200 μg or 800 μg) and was similar for the four ethnic groups: 80.8%–81.8% at the FF 200 μg dose and 81.8%–82.9% at the FF 800 μg dose.

The nominal ETD, calculated as the mass of the dose that has passed beyond the throat and therefore has the greatest potential to be available for lung deposition, accounted for approximately 22% of the nominal dose (200 μg or 800 μg) and was similar for the four ethnic groups: 20.4%–23.8% at the FF 200 μg dose and 19.5%–22.8% at the FF 800 μg dose. The mass of the emitted dose, with a particle size <2 μm accounted for approximately 5% of the nominal dose (200 μg or 800 μg) and was similar for the four ethnic groups: 5.1%–5.6% at the FF 200 μg dose and 3.8%–4.4% at the FF 800 μg dose.

## Discussion

The main objective of this open label, randomized, two way crossover study was to evaluate potential differences between East Asian populations (Chinese, Japanese and Korean) compared with Caucasian subjects on PK of the inhaled corticosteroid FF (repeat doses of 200 μg and 800 μg) when administered from the DPI, and when given as a single i.v. dose (FF 250 μg).

Following i.v. FF 250 μg, there was no evidence for differences for *V*_ss_ in Chinese, Japanese and Korean subjects compared with Caucasian subjects. There was no evidence for a difference between Japanese and Caucasian subjects whilst there was some evidence for slightly lower (<20%) plasma CL in the Chinese and Korean subjects compared with Caucasian subjects. However, these modest differences could be accounted for by the lower body weights in the East Asian populations, compared with the Caucasian subjects. FF is primarily metabolized by CYP3A4 and the similar i.v. PK for FF are consistent with the similar CYP3A4 activity seen across the four populations, as measured by both the urine cortisol : 6-β-hydroxy cortisol ratios and the plasma 4-β-hydroxy-cholesterol concentrations. The relatively small differences in the urine cortisol : 6-β-hydroxy cortisol ratio were considered to be unlikely to have any clinically meaningful effect given the high capacity of the CYP3A4 system and the low clinical doses of FF. All ethnic groups had large values of *V*_ss_, indicating extensive distribution of FF into tissues and high plasma CL which was consistent with previous PK data following i.v. administration [16].

There was evidence for higher systemic FF exposure following inhaled dosing with both 200 μg and 800 μg once daily in Chinese, Japanese and Korean subjects compared with Caucasian subjects that was not explained by body weight differences. At the 800 μg inhaled dose, where the PK data were more robust due to higher systemic exposure in all populations, the higher exposure in East Asian populations was reflected in higher overall inhaled bioavailability in the Chinese, Japanese and Korean subjects compared with Caucasian subjects. Pharyngometry and inhalation profiling data indicated that there was no evidence for marked differences between the ethnic groups in mouth and throat size or the peak inspiratory flow rate achieved. Any slight differences could be attributed to the difference in demographic attributes. Modelled and predicted total emitted dose, ETD and mass <2 μm as determined by the *in vitro* simulation method showed no marked differences between the ethnic groups, indicating that any difference in parameters of inhalation for the subjects in the study did not result in a difference in the predicted attributes of FF dose emission. Consequently, the higher systemic exposure to FF in the East Asian ethnic groups following inhaled dosing could not be attributed to differences in the modelled and predicted amount of FF delivered to the lung. The estimated ETD, when calculated as a percentage of the nominal dose (200 μg or 800 μg) was higher than the estimated values of absolute bioavailability in all ethnic groups and for both inhaled treatments. Since the oral bioavailability of the swallowed portion is negligible [12] this may be indicative that some of the dry powder dose delivered to the lung may be removed from the lung by mucociliary clearance prior to dissolution since FF is a poorly soluble molecule. Therefore, the higher bioavailability in East Asian subjects could indicate that a greater proportion of the dry powder ETD (estimated from the electronic lung prediction) reaches the systemic circulation, compared with Caucasian subjects.

In all four ethnic groups the *t*_1/2_ following inhaled administration of FF was notably longer than that seen following i.v. dosing. As previously reported (predominantly in Caucasian subjects) the elimination of FF is absorption rate limited following inhaled administration [17] and since oral bioavailability is in the region of 1% [12], the majority of exposure from inhaled dosing is due to absorption from the lung. The low oral bioavailability of FF is due to high first pass metabolism of absorbed drug by CYP3A4 and since this study has shown no notable difference in the activity of this enzyme in the East Asian and Caucasian subjects, similarly low oral bioavailability would be expected in East Asian subjects. To investigate the ethnic differences in FF PK further the inhaled time–concentration data were subjected to deconvolution analysis. Results from this analysis suggest that following inhaled administration FF resides in the lung of Chinese, Japanese and Korean subjects longer than for Caucasian subjects, and hence provides more opportunity for absorption and therefore greater bioavailability. FF absorption was essentially complete for all populations by the end of a dosing interval (24 h) and hence, significantly greater pulmonary accumulation of FF would not be anticipated in East Asian subjects compared with Caucasian subjects.

The reason for the longer residence time for FF in the lungs of subjects of East Asian ancestry is currently unknown. Although the reasons for the higher systemic FF exposure seen in East Asian subjects compared with Caucasians following inhaled dosing are not known this may be a consequence of differences in mucociliary clearance and/or differences in dissolution of FF in lung fluid (smaller lungs in East Asians). FF is not anticipated to be a substrate of anion or cation transporters and although FF is a substrate of the transporter P-glycoprotein, inter-ethnic differences in activity are not anticipated.

There were no major differences in the 24 h cortisol profiles between Caucasians and Japanese, Chinese or Koreans following 7 days of once daily inhaled FF 200 μg. The circadian rhythm for serum cortisol is well established. The healthy subjects in this study were domiciled within the clinical unit, with the time zero for serum cortisol sampling and time of dosing being the same for all subjects. Therefore, differential circadian rhythm would not be anticipated between the groups. This is reflected in Figure S2 and hence more sophisticated modelling of the serum cortisol data was not considered necessary to conduct the ethnic group comparisons. For all groups, trough serum cortisol concentrations were observed at 16 h (*circa* 00.00 h) whilst maximum serum cortisol concentrations were observed at 24 h (*circa* 08.00 h). There was no evidence for a difference in serum cortisol weighted mean between Caucasians and Chinese or Korean healthy subjects (average 8–10% difference) following 7 days of once-daily inhaled FF 200 μg. However, there was an average 22% (90% CI 12, 30%) lower serum cortisol weighted mean in Japanese subjects compared with Caucasian subjects. Despite this difference there was considerable overlap in the individual 24 h weighted mean serum cortisol values on day 7 (17/20 Japanese individual values were in the range for Caucasian subjects). In addition, there were no major differences between Japanese and Caucasian subjects in the trough serum cortisol concentration observed at 16 h, and although there was some difference in serum cortisol concentrations at 24 h between Japanese and Caucasian subjects on day 7, similar differences were seen on day −1 (i.e. pre-treatment). The relationship between cortisol suppression and FF AUC is well characterized and weighted mean AUC serum cortisol is a well-established endpoint for evaluation of hypothalamic-pituitary axis effects. The objective of the cortisol assessment in this study was to assess potential effects at the clinical dose rather than at the supra-therapeutic dose. At the 800 μg supra-therapeutic dose cortisol suppression would have been anticipated for all ethnic groups and there was not considered to be value in estimating this.

Although the FF systemic exposure was higher in the Chinese, Japanese and Korean subjects, the FF AUC(0,24 h) observed at FF 200 μg once daily was still considerably below levels of 1000 pg ml^−1^ h which is considered to be the threshold for significant cortisol suppression (a 20% reduction) in placebo controlled studies [18]. FF 200 μg was not associated with any effects on cortisol in a number of clinical studies [19,20]. A limitation of this study in assessing effect on cortisol is that it was a secondary endpoint in this study and the design was not optimized for measuring this parameter (e.g. it did not include a placebo crossover arm) and it is clear that there was a time effect in the study with a decline in cortisol concentrations between day −1 and day 7 in all ethnic groups, including Caucasians. For example, comparing geometric mean values on day 7 with day −1 there was a 17% drop from baseline in 24 h serum cortisol weighted mean for Caucasian subjects, a 21% drop from baseline in both Chinese and Korean subjects and a 32% drop from baseline in Japanese subjects. However, overall, if it is accepted that FF 200 μg does not reduce serum cortisol in Caucasian subjects, these data do suggest that there may be a slightly greater effect on cortisol in Japanese subjects at FF 200 μg, but data in the target patient population will be required to determine the clinical significance.

All FF treatments were safe and well tolerated with no marked quantitative or qualitative differences in safety endpoints, including incidence of EA reporting between the ethnic groups.

In conclusion, there were no marked differences in the inherent PK of FF following i.v. FF 250 μg between the ethnic groups other than those accounted for by body weight differences. FF systemic exposure was higher following inhaled dosing with both 200 μg and 800 μg once daily in Chinese, Japanese and Korean subjects compared with Caucasian subjects. Results from deconvolution analysis suggested that following inhaled administration FF resided in the lung of Chinese, Japanese and Korean subjects longer (average MAT approximately double) than for Caucasian subjects and hence provided opportunity for the greater bioavailability. Overall, the modestly higher (<50%) FF systemic exposure seen in East Asian subjects was not associated with a clinically significant effect on serum cortisol suggesting that a clinical dose adjustment in East Asian subjects is not required.

The study (protocol number HZA113477 [clinicaltrials.gov; NCT01000597]) presented in this manuscript was funded by GlaxoSmithKline. All listed authors meet the criteria for authorship set forth by the International Committee for Medical Journal Editors. All the authors are employees of GlaxoSmithKline. The authors wish to acknowledge Worldwide Bioanalysis DMPK (Ware, UK) for analyzing the PK samples, members of the GlaxoSmithKline R&D Inhaled Delivery Science team (Inhaled Product and Device Technology) for processing the pharyngometry and inhalation profiles and conducting the electronic lung experiments and the late Dr Patricia Burnell for her involvement in the protocol phase. Editorial support in the form of copyediting and graphic services was provided by David Cutler, PhD at Gardiner-Caldwell Communications (Macclesfield, UK) and was funded by GlaxoSmithKline.

## Competing Interests

All authors have completed the Unified Competing Interest form at http://www.icmje.org/coi_disclosure.pdf (available on request from the corresponding author). A. Allen, J. Bal, M. Hamilton, A. Cheesbrough and R. Kempsford are employees of and hold stock in GlaxoSmithKline.

## Notes

1 ELLIPTA™ is a registered trademark of the GlaxoSmithKline group of companies.

## Supporting Information

**Figure S1** Mean ± SD plasma semi-log concentration–time profiles for FF after administration of i.v. FF 250 μg. FF, fluticasone furoate; LLQ, lower limit of quantification; SD, standard deviation **Figure S2** Individual subject, adjusted geometric mean and 95% CI weighted mean serum cortisol (0–24 h) following 7 days repeat inhaled administration FF 200 μg. CI, confidence interval; FF, fluticasone furoate; WM, weighted mean **Table S1** Incidence of overall AEs and most frequently reported^a^ AEs by treatment regimen and by ethnic group
