# Supplementary material for: Pharmacokinetics and pharmacodynamics of intravenous and inhaled fluticasone furoate in healthy Caucasian and East Asian subjects
Source: Br J Clin Pharmacol. 2014 Apr 22;77(5):808–20. doi: 10.1111/bcp.12263 (PMC4004401; doi:10.1111/bcp.12263)

**Pharmacokinetics (PK) and pharmacodynamics (PD) of intravenous and inhaled fluticasone furoate (FF) in healthy Caucasian and East Asian subjects**

**Ann Allen,^1^ Joanne Bal,^2^ Anne Cheesbrough,^2^ Melanie Hamilton^3^ & Rodger Kempsford^1^**

**Supporting information**

**Ethnic origin definitions**

Caucasian: defined as having four grandparents who were descendents of European Caucasians).

Japanese: defined as being born in mainland Japan, having four ethnic Japanese grandparents, holding a Japanese passport or identity papers and being able to speak Japanese).

Korean: defined as being born in mainland Korea, having four ethnic Korean grandparents, holding a Korean passport or identity papers and being able to speak Korean).

Chinese (defined as being born in mainland China or Hong Kong, having four ethnic Chinese grandparents, holding a Chinese passport or identity papers and being able to speak Chinese).

Chinese, Japanese and Korean subjects were required to have lived outside their respective countries for less than 10 years.

**Details of study/clinic visits, timing of assessments, restrictions related to food, drink and exercise intake, and assessment of treatment compliance**

The inhaled treatment period was conducted in two parts (Part A and Part B).

*Part A.*

Each subject attended the unit for a short visit on the morning of Day –2 (i.e. 2 days before the start of the treatment phase) to provide clinical laboratory safety samples. Subjects were then admitted to the clinical unit on the evening of Day
–2 and remained in the unit until approximately 1 h post-dose on Day 1. Subjects self-administered their doses at home on Days 2–6. Subjects returned to the unit on the evening of Day 6 and remained in the unit until the morning of Day 8, when all assessments at 24 h post-Day 7 dose had been completed.

*Part B.*

Each subject attended the unit for a short visit on the morning of Day –1 to provide clinical laboratory safety samples. Subjects were then admitted to the clinical unit on the evening of Day –1, and remained in the unit until all assessments had been completed at 24 h after the Day 1 dose. Subjects returned to the unit to provide samples for PK analysis at 32 h and 48 h after the Day 1 dose. No dose was given on Day 2 to allow the PK of single dose FF 800 μg to be evaluated. Following collection of the 48 h PK sample subjects self-administered the Day 3 dose within the clinical unit, and self-administered FF at home on days 4–7. Subjects returned to the clinical unit on the evening of Day 7, received their final dose on the morning of Day 8 and remained there until all assessments at 24 h after the Day 8 dose had been completed. Subjects returned to the unit for PK sampling at 32, 48 and 72 h following the Day 8 dose. Subjects were discharged from the unit following the 72 h sample collection.

In both parts, subjects were required to fast from midnight before each full in-house assessment day until 4 h post-dose, with the exception of water which was allowed freely except for 1 h either side of dosing. Restrictions were placed on strenuous exercise and on the consumption of grapefruit, Seville oranges, caffeine, alcohol and tobacco. Subjects had to follow protocol-defined contraception requirements throughout the study. When subjects self-administered at home the time and date of each dose was recorded in a diary. When the subject returned to the site the pharmacist or study nurse made a note of the dose count on the dose counter of the inhaler and recorded on a drug accountability log. This was checked by the study monitor and recorded on the electronic case report form.

**Venous blood sampling times for analysis of plasma drug concentration**

Inhaled FF 200 μg on Day 7: pre-dose, 15, 30 and 45 min, and 1, 1.5, 2, 3, 4, 6, 8, 10, 12, 16 and 24 h after dosing.

Inhaled FF 800 μg on Day 1 and Day 8: pre-dose and at 15, 30 and 45 min, and 1, 1.5, 2, 3, 4, 6, 8, 10, 12, 16, 24, 32 and 48 h after the start of dosing. A sample was also collected at 72 h post-dose for FF 800 μg Day 8 only.

IV FF dosing: pre-dose and at 10, 20, 25, 30 and 45 min and 1, 1.5, 2, 3, 4, 6, 8, 10, 12, 16, 24, 32 and 48 h after the start of dosing.

**Analytical methods for plasma sample analyses**

For the solid phase extraction followed by high performance liquid chromatography with tandem mass spectrometry, a gradient system using 5 mM ammonium formate and methanol was run with column ACE 50 *2.1 mm, C18 3 μm, and Hichrom Ltd running at 45ºC. The ion transition for FF was m/z 539 to 313. Where reported concentrations were above the higher limit of quantification the plasma samples were diluted, as appropriate, prior to analysis to provide concentrations within the validated range. Quality controls prepared at three different concentrations were analyzed with each batch of samples against separately prepared calibration standards to assess the day-to-day performance of the assay. Quality control results from this study met the acceptance criteria of no more than one-third of the quality control results deviating from the nominal concentration by more than 15%, with at least one quality control result acceptable at each concentration.

**Serum cortisol analyses**

On Day –1 serum cortisol samples were collected according to the same schedule as on Day 7, with the zero time point for these samples being between 7.00 am and 9.00 am on Day –1, and all other time points were relative to the Day –1 zero time point. Serum samples obtained by centrifugation were transferred, frozen, and stored frozen at –20ºC until shipment. Serum samples were analyzed for cortisol (100 µl of serum) with a validated analytical method using high throughput liquid chromatography with tandem mass spectrometry and a Thermo electron TSQ Quantum. The validation range of the assay was 0.1–100 µg/dl for cortisol, and the lower limit of quantification for cortisol was 0.1 µg/dl. Within-run precision, between-run precision and bias were all ≤20%. Where reported concentrations were above the higher limit of quantification the serum samples were diluted, as appropriate, prior to analysis to provide concentrations within the validated range. Quality controls prepared at three different concentrations were analyzed with each batch of samples against separately prepared calibration standards to assess the day-to-day performance of the assay. Quality control results from this study met the acceptance criteria of no more than one-third of the quality control results deviating from the nominal concentration by more than 20%, with at least one quality control result acceptable at each concentration.

Supporting Table 1 Incidence of overall AEs and most frequently reported^a^ AEs by treatment regimen and by ethnic group

| **Treatment** | **AE** | **Caucasian**  **subjects**  **N = 19**  **(%)** | **Chinese  subjects**  **N = 20**  **(%)** | **Japanese**  **subjects**  **N = 20**  **(%)** | **Korean  subjects**  **N = 20**  **(%)** |
| --- | --- | --- | --- | --- | --- |
| Inhaled FF 200 mcg | Any AE | 42 | 60 | 45 | 5 |
|  | Headache | 16 | 15 | 15 | 0 |
|  | Diarrhoea | 0 | 0 | 10 | 0 |
|  | Abdominal pain | 0 | 0 | 10 | 0 |
|  | Oropharyngeal pain | 0 | 0 | 15 | 0 |
| Inhaled FF 800 mcg | Any AE | 50 | 70 | 30 | 25 |
|  | Headache | 28 | 25 | 5 | 10 |
|  | Excoriation | 11 | 0 | 0 | 0 |
|  | Lethargy | 0 | 10 | 0 | 0 |
|  | Diarrhoea | 0 | 0 | 10 | 0 |
|  | Oropharyngeal pain | 17 | 20 | 5 | 0 |
| Intravenous FF 250 mcg | Any AE | 42 | 35 | 21 | 20 |
|  | Headache | 32 | 5 | 11 | 0 |
|  | Catheter site pain | 11 | 15 | 0 | 0 |

^a^AEs reported by more than one subject per ethnic group

AE, adverse event; FF, fluticasone furoate.

**Supporting Figure 1** Mean+/-SD plasma semi-log concentration-time profiles for FF after administration of intravenous FF 250 μg.

FF, fluticasone furoate; LLQ, lower limit of quantification; SD, standard deviation.

**
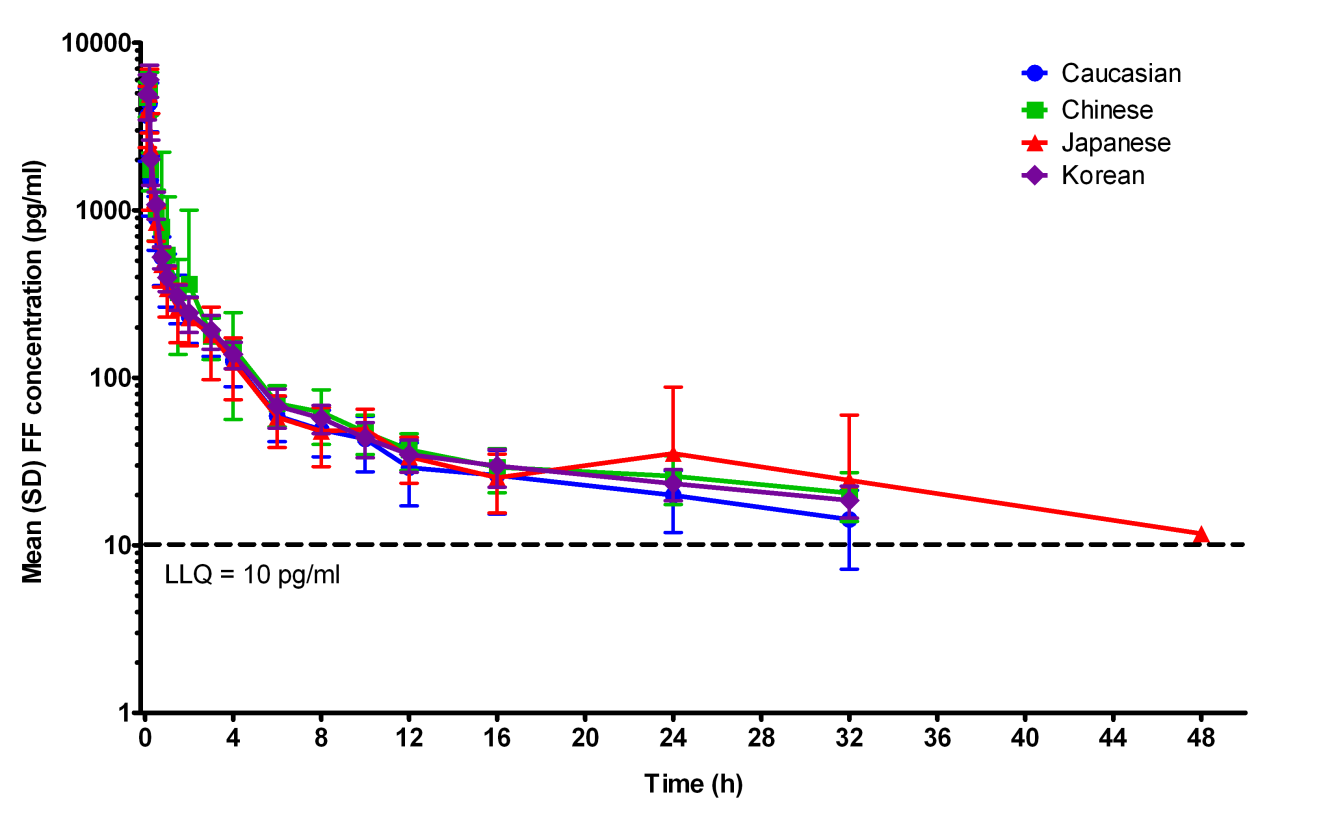
**

**Supporting Figure 2** Individual subject, adjusted geometric mean and 95% CI weighted mean serum cortisol (0–24 h) following 7 days repeat inhaled administration FF 200 μg.

CI, confidence interval; FF, fluticasone furoate; WM, weighted mean.


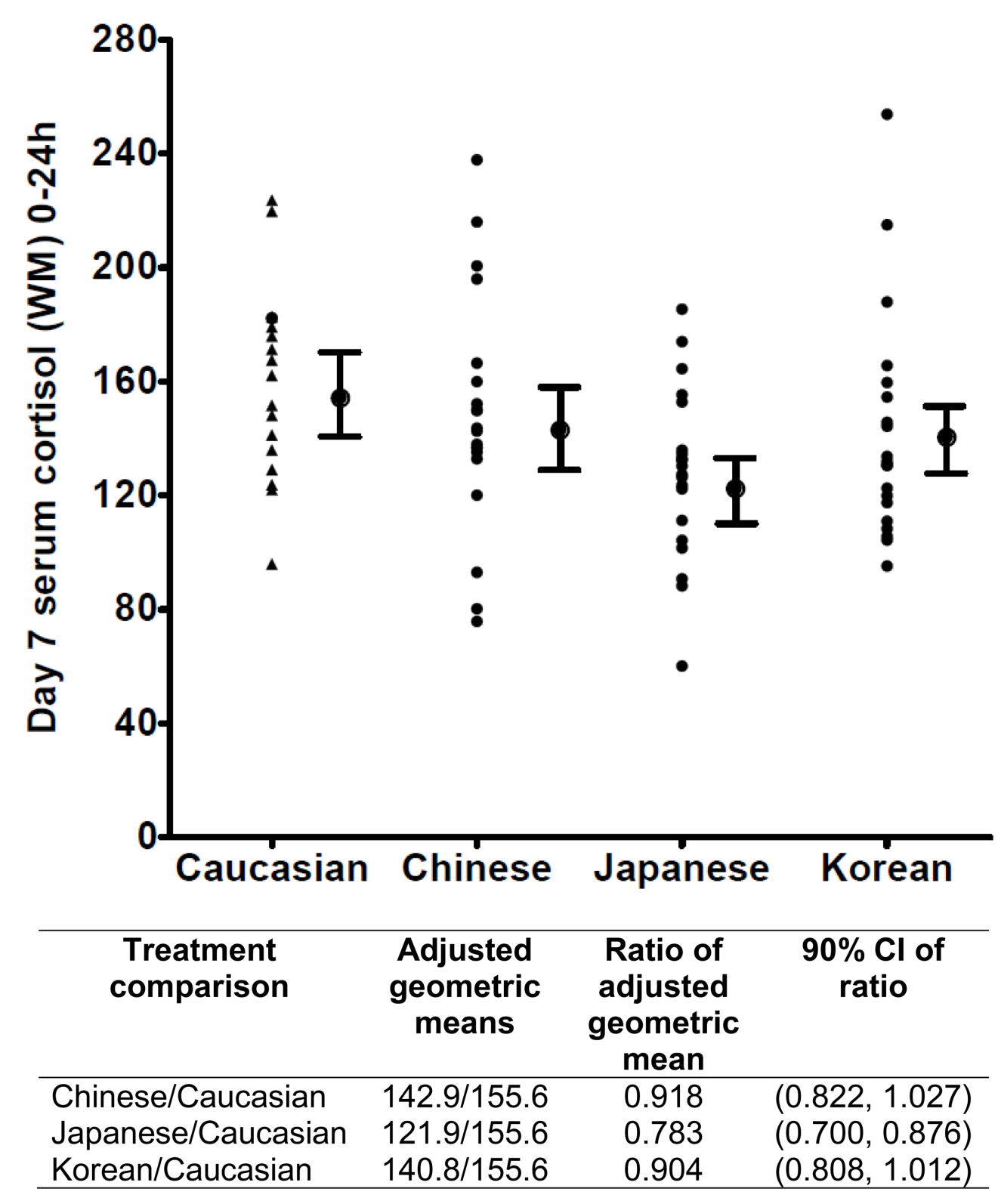

Supplement: Supplementary file 1 — Figure S1 Mean ± SD plasma semi-log concentration–time profiles for FF after administration of i.v. FF 250 μg. FF, fluticasone furoate; LLQ, lower limit of quantification; SD, standard deviation Figure S2 Individual subject, adjusted geometric mean and 95% CI weighted mean serum cortisol (0–24 h) following 7 days repeat inhaled administration FF 200 μg. CI, confidence interval; FF, fluticasone furoate; WM, weighted mean Table S1 Incidence of overall AEs and most frequently reporteda AEs by treatment regimen and by ethnic group [file bcp0077-0808-sd1.docx]
